# Spinal cord ischemia following cervical transforaminal epidural steroid injection

**Published:** 2014-10

**Authors:** Tahir H. Obeid, Mohamed A. Gornas, Ayman A. El-Mesallamy

**Affiliations:** *From the Departments of Neurology (Obeid, Gornas), and Radiology (El-Mesallamy), Alzaytouna Specialist Hospital, Khartoum, Sudan*

Transforaminal cervical epidural steroid injection (TFCESI) is a common non-surgical adjunct in the management of patients with cervical radiculopathy. It has gained popularity over the interlaminar approach because of its direct delivery of the steroid into the site of pathology. These procedures require fluoroscopic guidance in addition to digital subtraction angiography (DSA) as they provide direct visualization of the site of treatment. Several complications have been attributed to these injections including increased pain at injection site, increased radicular pain, lightheadedness, increased spine pain, non-specific headache, and nausea. However, numerous reports have described devastating complications related to these injections.^1^,^2^ We report a transient neurologic sequelae in a patient that underwent a cervical epidural injection without fluoroscopic guidance.

**Figure 1:**
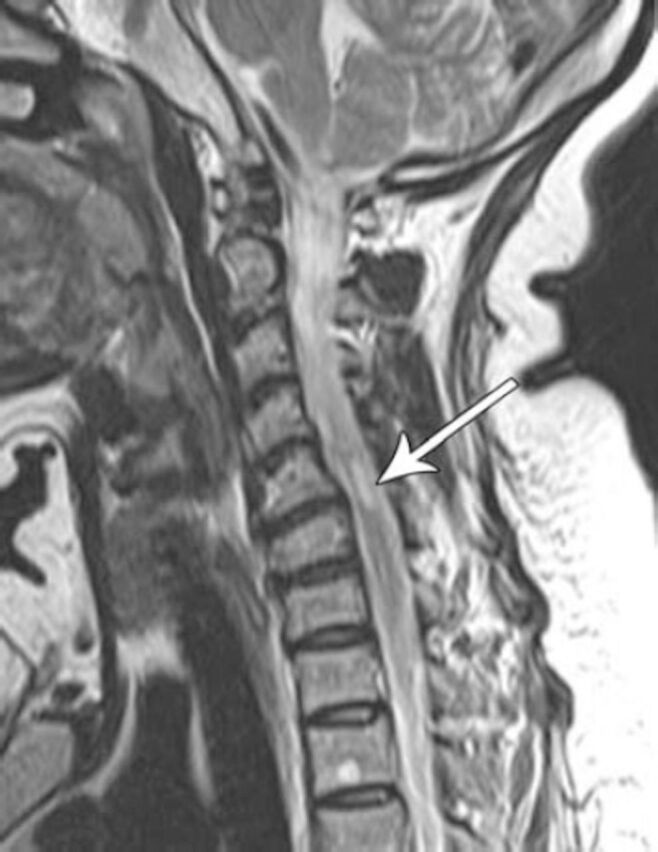
Sagittal T2 MRI of the cervical spine of a 38-year-old female who underwent an epidural steroid injection of the neck showing a right-sided intramedullary area of abnormal signal at the C5-6 level.

A 38-year-old Sudanese female underwent a TFCESI for cervical C5 radiculopathy of the right upper limb in a private hospital in Khartoum, Sudan. She was seen in our hospital 2 weeks prior to the procedure for radicular pain without any associated neurological deficit. The procedure was not carried out under fluoroscopic guidance or DSA. No details of the procedure were obtained. On the day of the procedure, she developed right shoulder weakness with mild heaviness of the right lower limb. She presented to our facility 2 days later and had no improvement of her symptoms. Neurological examination revealed evidence of cervical myelopathy evident by paraparesis of the lower limbs with power grade 4 (MRC), the right being more affected than the left. Lab investigations including complete blood count, screening for connective tissue diseases, and CSF for oligoclonal bands were all normal. The brain MRI revealed no abnormality. However, the MRI of the cervical spine demonstrated an intramedullary plaque like-lesion of abnormal signal intensity corresponding to the C5-6 region (**Figure 1**). She was initially treated with steroids as the initial impression was of an underlying demyelinating disease. She showed complete recovery of her symptoms after 6 weeks follow-up, except for impaired sensations of the C5 dermatome of the right side and power of grade 4+.

Epidural steroid injections (ESI) are a widely used procedure for the management of low back and neck pain with and without radiculopathy.^3^ Patients are usually injected with corticosteroids into the epidural space, where they are believed to have an anti-inflammatory effect on the spinal nerve.^3^ Several trials show moderate benefit from these injections; however, there is still controversy concerning their long-term effect.^4^ In our case, the patient developed acute transient paraparesis of the limbs, more on the right side. She showed marked improvement in the following weeks. The cervical MRI revealed an abnormal signal intensity corresponding to the C5-6 region. This could be explained by the fact that the cord infarction was only partial with ischemia occurring to the small branches of the radicular arteries. The cause of ischemia could be due to direct injection of the drug into the radicular arteries as the procedure was carried out blindly or as a result of embolization caused by particulates from the steroid used. It is unlikely to be clinically isolated syndrome or multiple sclerosis in the absence of supportive evidence, and ischemia of the cord is the most likely explanation.

Although TFCESI is considered safe, there are no trials supporting safety or efficacy.^3^ They are associated with rare, but devastating complications including paralysis, brainstem infarction, epidural lipomatosis, and epidural abscess.^1^,^2^ There is a significant risk of serious neurologic injury following TFCESI. The causes being either an embolic effect or intra-arterial injection of a steroid causing a distal infarct. A recent outbreak of fungal meningitis was reported due to the use of infected corticosteroids leading to 750 cases of fungal infection and 64 reported deaths.^5^

The incidence of these rare complications remains unknown, though these procedures under fluoroscopic guidance and DSA may reduce these complications. Both patients and practitioners alike should be aware of the serious risks associated with these injections. We emphasize that TFCESI should be carried out under fluoroscopic guidance with control enhancement with DSA.
